# A Modular Assembly of Spinal Cord–Like Tissue Allows Targeted Tissue Repair in the Transected Spinal Cord

**DOI:** 10.1002/advs.201800261

**Published:** 2018-07-20

**Authors:** Bi‐Qin Lai, Bo Feng, Ming‐Tian Che, Lai‐Jian Wang, Song Cai, Meng‐Yao Huang, Huai‐Yu Gu, Bing Jiang, Eng‐Ang Ling, Meng Li, Xiang Zeng, Yuan‐Shan Zeng

**Affiliations:** ^1^ Key Laboratory for Stem Cells and Tissue Engineering (Sun Yat‐sen University) Ministry of Education Guangzhou 510080 China; ^2^ Department of Histology and Embryology Zhongshan School of Medicine Sun Yat‐sen University Guangzhou 510080 China; ^3^ Co‐Innovation Center of Neuroregeneration Nantong University Nantong 226001 China; ^4^ Guangdong Provincial Key Laboratory of Brain Function and Disease Zhongshan School of Medicine Sun Yat‐sen University Guangzhou 510080 China; ^5^ Department of Human Anatomy Zhongshan School of Medicine Sun Yat‐sen University Guangzhou 510080 China; ^6^ Department of Anatomy Yong Loo Lin School of Medicine National University of Singapore Singapore 117594 Singapore; ^7^ Neuroscience and Mental Health Research Institute School of Medicine Cardiff University Cardiff CF24 4HQ UK; ^8^ Institute of Spinal Cord Injury Sun Yat‐sen University Guangzhou 510120 China

**Keywords:** organoids, spinal cord injuries, spinal cord–like tissues, tissue engineering, transplantation

## Abstract

Tissue engineering–based neural construction holds promise in providing organoids with defined differentiation and therapeutic potentials. Here, a bioengineered transplantable spinal cord–like tissue (SCLT) is assembled in vitro by simulating the white matter and gray matter composition of the spinal cord using neural stem cell–based tissue engineering technique. Whether the organoid would execute targeted repair in injured spinal cord is evaluated. The integrated SCLT, assembled by white matter–like tissue (WMLT) module and gray matter–like tissue (GMLT) module, shares architectural, phenotypic, and functional similarities to the adult rat spinal cord. Organotypic coculturing with the dorsal root ganglion or muscle cells shows that the SCLT embraces spinal cord organogenesis potentials to establish connections with the targets, respectively. Transplantation of the SCLT into the transected spinal cord results in a significant motor function recovery of the paralyzed hind limbs in rats. Additionally, targeted spinal cord tissue repair is achieved by the modular design of SCLT, as evidenced by an increased remyelination in the WMLT area and an enlarged innervation in the GMLT area. More importantly, the pro‐regeneration milieu facilitates the formation of a neuronal relay by the donor neurons, allowing the conduction of descending and ascending neural inputs.

## 1 Introduction

Spinal cord injury (SCI) causes massive death of neurons and glia, leaving a hostile microenvironment that impedes nerve fiber regeneration and tissue repair.^1^ Although endogenous neural stem cells (NSCs) have the potential for spinal repair, their bias toward astrocytic differentiation hinders the restoration of lost neurons and oligodentrocytes. Moreover, astrocyte differentiation of NSCs also contributes to glial scar after spinal cord injury.^2^, ^3^ Yet, thus far, there is no effective treatment to this condition. The recent development of stem cell–based tissue engineering technology has provided a promising strategy for treating SCI.^4^ With the insights gained from developmental biology and the advancement in 3D culturing, tissue engineering has entered a new era of constructing of multiple tissue types or organoids.^5^ It is believed that tissue engineering would help realize constructing a functional tissue or organoid through the combination of stem cells, functional biomaterials, and neurotrophic factors to treat diseases of the central nervous system, including SCI.^6^

It has been reported that significant motor sensory recovery may be achieved when donor NSCs were chemically induced into neurons in vivo to reconnect the neural pathways of the injured spinal cord.^7^ It is believed that the exogenous stem cell–derived neurons can contribute to a newly established “neuronal relay” that would reform the synaptic connection of the interrupted neural pathway and reconstruct the neural transmission circuit. Thus, the use of “neuronal relay” strategy to repair the injured spinal cord has received increasing recognition^2^, ^8^ in recent years. When stem cells were combined with biomaterials supporting sustained release of neurotrophic factors, it is believed that the postinjury microenvironment could be improved, allowing longer axonal growth and more synaptic connections with the host neurons.^9^ However, safety issue remains a major concern for direct transplantation of stem cells into the spinal cord because of the possibility of migration and ectopic proliferation, and the uncertainty of the differentiation of the grafted cells.^10^

In our previous study, we have adopted the “tissue engineering neuronal relay” strategy to repair the rat SCI by tissue engineering approach. To strengthen the safety, the stem cells were induced into young neurons in vitro, prior to transplantation.^11^ The preconstructed functional neuronal network had provided a favorable microenvironment that increased the survival of donor cells, thereby fostering the regeneration of the host nervous tissue.^11^, ^12^ More importantly, this in vitro tissue construction strategy is believed to minimize the uncertainty of stem cell differentiation in vivo.[[qv: 6b]] Using the same tissue engineering strategy, we have also constructed NSC‐derived oligodendrocytes that formed distinct multilayered lamellae both in vitro and in vivo.^13^ These two tissue construction strategies have allowed the donor cells to differentiate with specific function for repair of injured spinal cord. Buoyed by these advancements in spinal cord tissue engineering, it is presumable that a spinal cord organoid that recapitulates the major morphological and functional properties of both white and gray matters would provide reinforced therapeutic efficacy in SCI repair, but such configuration has not yet been achieved.

In the present study, we have combined the neuronal and oligodendroglial induction techniques and developed a novel spinal cord–like tissue (SCLT) in vitro by modular assembly of the white matter–like tissue (WMLT) and the gray matter–like tissue (GMLT) from NSCs with structural and functional simulation to the rat spinal cord. The neural cells in the SCLT exhibited targeted repair in the injured white matter and gray matter, respectively; moreover, both the repaired white matter and gray matter functioned synergistically to rebuild the neural pathway and to improve paralysis hind limb motor function of rats after SCI. It is also envisaged that the SCLT would serve as a useful in vitro platform for future study of neuropharmacology and neurodevelopment of the spinal cord.

## 2 Results

### 2.1 Modular Assembly of SCLT

**Figure 1:**
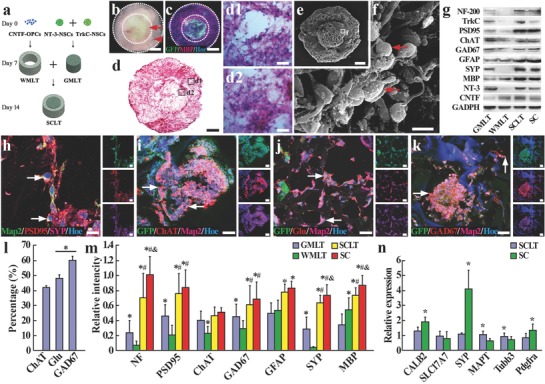
Assembly of the SCLT and its phenotypic similarity to normal adult spinal cord. a) A schematic diagram of the assembling process of a SCLT. b) An overview of the SCLT showing good integration of the WMLT (arrowhead) and the GMLT (arrow) with no obvious gap. c) Distinct partition of the GMLT module (GFP positive) and the WMLT module (GFP negative, MPB positive) after being assembled into a SCLT for 7 days. d,d1,d2) H&E staining of a SCLT showing good cell distribution in both WMLT and GMLT region. e,f) Surface view of a SCLT as seen by scanning electron microscopy. g) After 14 days of culturing, protein expression of individually cultured GMLT or WMLT, SCLT, and normal spinal cord (SC). h) Cells (arrows) in the GMLT were triple‐stained for Map2, PSD95, and SYP. GFP and Map2 positive cells in the GMLT expressed i) ChAT, j) glutamate (Glu), or k) GAD67. l) Bar chart showing the percentage of ChAT, Glu, and GAD67 positive cells among all GFP positive cells. Asterisks indicate *p* < 0.05. m) Relative expression levels of different proteins in the GMLT, WMLT, SCLT, and SC groups, and asterisks, #, and & symbols indicate *p* < 0.05 when the SC group was compared with the GMLT, WMLT, or SCLT groups, respectively. n) Q‐PCR was used to detect the difference in expression of specific mRNA between the SCLT and normal spinal cord. Asterisks indicate *p* < 0.05 when the SC group was compared with the SCLT group. Scale bars = 500 µm in panels (b)–(d) and (e), 40 µm in panels (d1) and (d2), 5 µm in panel (f), 10 µm in panel (h), 50 µm in panels (i)–(k).

Through a combination of gene modification, 3D culture technique, and a modular assembly protocol (**Figure** **1**a), NSCs were induced in the SCLT into the major cell types (neuron, oligodendrocyte, and astrocyte) in the gray and white matters of the spinal cord. The GMLT module was constructed by promoting neuronal differentiation of neurotrophin‐3 (NT‐3)/TrkC gene–modified NSCs in the collagen sponge column, while the WMLT module was constructed by inducing the maturation of the ciliary neurotrophic factor (CNTF) gene–modified oligodendrocyte precursor cells (OPCs) derived from NSCs in the collagen sponge ring (Figure 1a). After separated culturing for 7 days, the two modules were assembled into one entity, which was allowed to continue culturing for another 7 days for further maturation and mutual interaction (Figure 1a). Immunocytochemistry was used to validate the expression of anticipated markers during stepwise construction of the modular assembly and maturation process by cells in the GMLT, WMLT, and SCLT after 14 days of culture. Nestin positive NSCs from the neurospheres (Figure S1a, Supporting Information) were transfected with a vector to either express NT‐3 or its receptor TrkC, and then seeded in a 3D collagen sponge column for 7 days of culture for the construction of GMLT (Figure S1b, Supporting Information). Immunocytochemical analysis revealed that ≈70% of the GMLT cells expressed the neuronal marker Map2, and 20% of the cells expressed the astrocyte marker glial fibrillary acidic protein (GFAP) at this time point (Figure S1c, Supporting Information). Meanwhile, NSCs were induced into OPCs with exogenous triiodothyronine (T3) and platelet‐derived growth factor (PDGF), and the induced cells showed a high purity of neuroglycan 2 (NG2, a marker for OPCs, Figure S1d, Supporting Information). The OPCs were induced for differentiation to construct the WMLT. After 7 days of culture in the collagen sponge ring, the cells were able to differentiate into myelin basic protein (MBP) positive cells with ubiquitous expression of CNTF (Figure S1e, Supporting Information). It is estimated that 80% of the differentiating cells expressed oligodendrocyte marker MBP, and 20% of them expressed GFAP at this time point (Figure S1f, Supporting Information).

Thus, following 14 days culturing in vitro, the assembled SCLT roughly simulated the anatomical partitions of a spinal cord, i.e., the white matter and the gray matter (Figure 1b–d). The assembly exhibited a good integration with no discernible gap between the two modules (Figure 1b, phase‐contrast imaging of a SCLT in the culture dish). Additionally, cells seeded in each module did not seem to migrate significantly into the other module at this time point, as shown in Figure 1c, where most of the cells in the GMLT (from green fluorescent protein (GFP) transgenic Sprague‐Dawlay (SD) rats) or the counterparts in the WMLT (from non‐GFP donor, immunostained by MBP, a myelin marker) remained within the same module. Hematoxylin and eosin (H&E)‐stained section sampled from the center of a serial of SCLT transverse sections showed the dense population of cells in both WMLT and GMLT modules (Figure 1d). A closer look from this section revealed good cell viability and enriched cell contacts (Figure 1d1,d2). Scanning electron microscopy (SEM) presented the surface view of a SCLT (Figure 1e). Higher magnification image showed the abundant cell contacts amid the dense cell population in the GMLT (Figure 1f).

### 2.2 Phenotypic Similarities between the SCLT and Adult Spinal Cord Tissue

Following 14 days single culture of GMLT or WMLT, or 7 days culture after they were assembled to SCLT, protein expression profile was measured by Western blotting. The results showed that the GMLT predominantly presented neuronal phenotypes, as demonstrated by the expression of a battery of molecular markers that include neurofilament (NF) as a pan mature neuronal marker, postsynaptic density protein 95 (PSD95), and synaptophysin (SYP) as post‐ and presynaptic markers, respectively, and neurotransmitter synthesizing proteins such as choline acetyltransferase (ChAT), glutamate decarboxylase 67 (GAD67). In contrast, the WMLT were enriched with cells expressing MBP, a myelin marker (Figure 1g,m). When the two modules were assembled into SCLT, the protein expression profile showed a high similarity to that of a mature spinal cord (a positive control) in markers of NF, PSD95, ChAT, GAD67, GFAP, SYP, and MBP, except for the exogenous genes like TrkC, NT‐3, and CNTF (Figure 1g,m).

After the assembly of SCLT, Map2 positive neurons in the GMLT region exhibited intense expression of PSD95 and SYP, suggesting the establishment of synaptic connections between neurons within SCLT (Figure 1h). Moreover, the detection of neurotransmitter glutamate (Glu) or neurotransmitter synthesizing enzymes ChAT and GAD67 in Map2 positive neurons indicated the neurochemical divergence following the neuronal induction (Figure 1i–k). Using quantitative real‐time polymerase chain reaction (Q‐PCR), the messenger RNA (mRNA) expression level for neuron and oligodendrocyte in SCLT after 14 days of culture resembled most of the phenotypes for terminal differentiation, despite a few immature ones, when compared with the normal adult rat spinal cord (SC, Figure 1n).

### 2.3 Myelination, Vesicle Releasing, and Neuronal Electrophysiological Activities inside the SCLT

**Figure 2:**
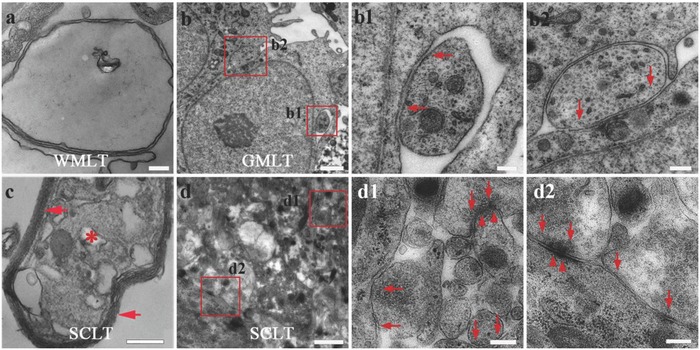
Myelin and synaptic formation potential of the SCLT. a,b,b1,b2) The singly cultured WMLT or GMLT module presents features of immature myelin sheath (a), or synaptic connections (arrows indicate small and dense vesicles in (b), (b1), (b2)) under the electron microscope, respectively. c) The SCLT shows multilamellar myelin sheaths (arrows in (c)) enwrapping an axonal profile (asterisk in (c)) with (d), (d1), (d2) seemingly more mature features of synapses, like the presynaptic vesicles (arrows in (d1), (d2)) and the postsynaptic membrane density (arrowheads in (d1), (d2)). Scale bars = 200 nm in panels (a), (b1), (b2), (c), (d1), and (d2), 1 µm in panels (b) and (d).

After 14 days single culture of GMLT or WMLT, or 7 days culture after they were assembled to SCLT, the samples were prepared for morphological and functional assessments as follows. By electron microscopy, thin myelin laminae were observed in the WMLT even without being assembled with GMLT, after 14 days single culturing (**Figure** **2**a). It is noteworthy that myelination within the WMLT may be independent from the axon since some myelin sheaths were devoid of axonal profile (Figure 2a). The neurons inside the 14 days singly cultured GMLT appeared to make many contacts with the neighboring cells (Figure 2b). Some of these intercellular contacts resembled an immature synapse feature, as indicated by the existence of small and dense vesicles detected inside the presynaptic component; however, postsynaptic density (PSD) in the postsynaptic component was not evident (Figure 2b1,b2). When the WMLT and the GMLT were assembled into the SCLT, multilayered myelin sheaths enwrapping an axonal profile were observed in the WMLT (Figure 2c). Moreover, more mature synaptic features, such as an increased number of synaptic vesicles at the axon terminals, and the formation of PSD at the postsynaptic component, were detected in neurons within the GMLT region (Figure 2d,d1,d2).

**Figure 3:**
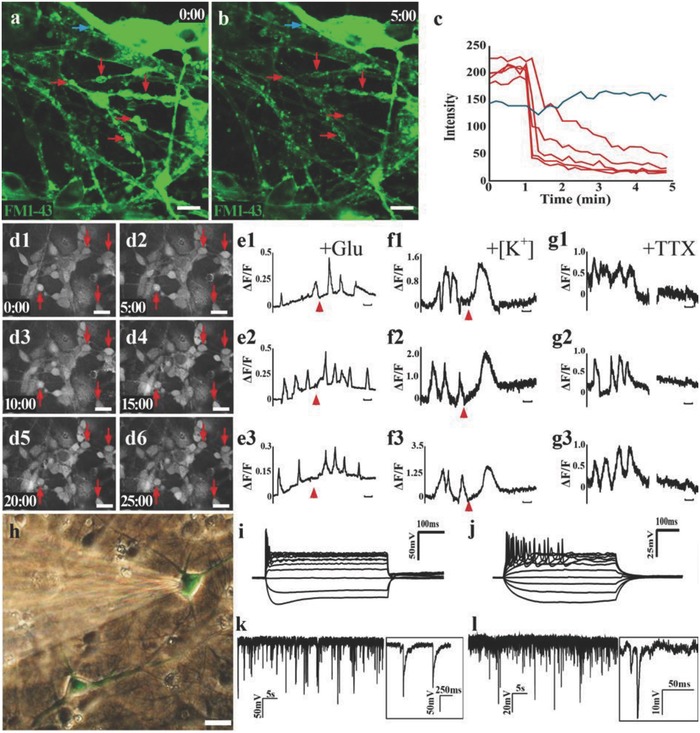
Functional assessment of the SCLT. a,b) Neurons in the GMLT were observed to unload prelabeled FM1‐43 dye (green) following membrane depolarization triggered by high [K^+^] stimulation, as shown by (c) the steep drops of fluorescence intensity after the stimulation. d1–d6) Neurons in the SCLT show spontaneous calcium surges (arrows) during Fluo‐4 calcium live cell imaging. Single‐cell tracing of calcium surges reveals that calcium activities in the neurons can be excited by (e1)–(e3) glutamate or (f1)–(f3) high [K^+^], and suppressed by (g1)–(g3) tetrodotoxin (TTX). h) Whole‐cell patch clamp recording exhibited the increased firing of action potentials of neurons from the culturing time (i) 7 days to (j) 14 days after the SCLT were assembled. k,l) High‐frequency miniature excitatory postsynaptic current (mEPSC in (k)) or miniature inhibitory postsynaptic current (mIPSC in (l)) were detected in neurons in the SCLT. Scale bars = 10 µm in panels (a), (b), and (d1)–(d6), 100 s in panels (e1)–(g3).

To further assess the functionality of neurons in the SCLT, we performed FM1‐43 dye releasing assay, calcium imaging, and patch clamp recordings. We found that SCLT neurons could internalize the FM1‐43 fluorescent dye following an initial dose of high [K^+^] and release it after a second dose (**Figure** **3**a–c). This is consistent with the fashion of mature neurons in FM dye loading and releasing following high [K^+^] stimulation.^14^ Ca^2+^ oscillations activity could be detected when neurons are loaded with calcium dye.^15^ Neurons in the SCLT presented spontaneous calcium surges in individual cells (Figure 3d1–d6). Transient increase of calcium surges was observed following an excitatory glutamate (Figure 3e1–e3) or high [K^+^] stimulation (Figure 3f1–f3). The spontaneous calcium surges could be blocked by tetrodotoxin (TTX, Figure 3g1–g3), suggesting that TTX‐sensitive voltage‐gated sodium channels, which contribute to the forming of action potentials in mature neurons,^16^ might exist in the neurons in the SCLT as well. On the other hand, neurons in the singly cultured GMLT did not present vesicle releasing capability following high [K^+^] stimulation nor Ca^2+^ oscillations activity, suggesting that they may not resemble mature neuronal properties (Figure S2, Supporting Information). In light of these findings, patch clamp experiment was carried out to study the electrophysiological properties of the neurons in the SCLT. We found that the capability of firing action potential strings by the neuron was increased when the culturing time of the SCLT was extended from 7 days (Figure 3i) to 14 days after the assembly (Figure 3j). Meanwhile, miniature excitatory postsynaptic currents (mEPSCs) and miniature inhibitory postsynaptic currents (mIPSCs) were also detected in the neurons inside the SCLT, given 14 days of culturing time (Figure 3k,l).

### 2.4 Structural Integration between the SCLT and Organotypic Dorsal Root Ganglion (DRG) or Muscle Cells

**Figure 4:**
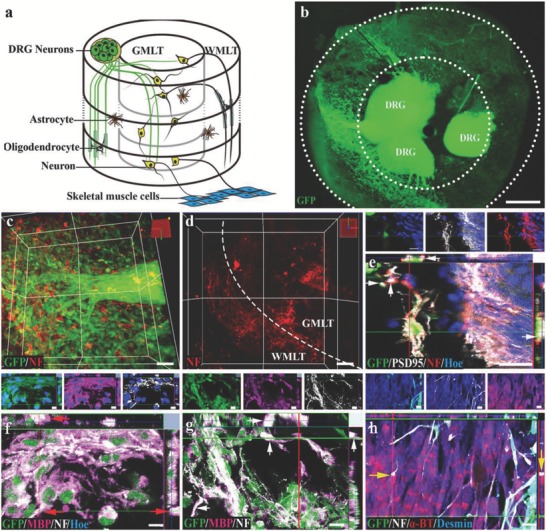
The SCLT establishes connections with DRG or muscle cells. a) A schematic diagram showing a SCLT establishes connections with the organotypic cultured DRG or muscle cells. b) GFP donor–derived DRG extended NF positive neurites into the c,d) SCLT. e) The neurons in GFP donor–derived GMLT region form close contacts with DRG (from GFP negative donor), with PSD95 expression at the contact sites. f,g) GFP donor derived WMLT of a SCLT‐expressed MBP (arrows) wrapping NF‐positive DRG neurites (GFP negative in (f)) or neurites (GFP negative in (g)) extending from the neurons in the GMLT region. h) The outgrowing NF positive neurites from GFP positive SCLT form close contacts with α‐BT‐expressing muscle cells with bouton‐like enlargement (arrows) at the terminal. Scale bars = 500 µm in panel (b), 50 µm in panels (c) and (d), 20 µm in panels (e)–(h).

We then investigated the potential of SCLT to establish synaptic connections with peripheral nerve cells or muscular junctions by organotypic cocultures with DRG or muscle cells, respectively (**Figure** **4**a). To better illustrate the relationship between the SCLT and the organotypic DRG, we have designed the experiment as follows:

1) DRGs (derived from GFP transgenic SD rats) + SCLTs (from wild‐type SD rats). This part was to study the innervations of DRGs into the SCLTs (Figure 4b–d).
2) SCLTs (GFP) + DRGs (wild type). This part was to study the interactions between nerve fibers from DRGs and SCLTs (Figure 4e).
3) SCLTs (with the WMLTs from GFP and the GMLTs from wild type) + DRGs (wild type). This part was to study the interactions between nerve fibers and myelin (Figure 4f,g).

Following 7 days of coculturing, DRG neurites (GFP positive, Figure 4b) were observed in the SCLT block (Figure 4c,d). The neurites from the DRG neurons (GFP negative) were in contacts with nerve fibers emanated from neurons of the SCLT (GFP positive). The expression of postsynaptic marker PSD95 in the latter may indicate the potential of synapse forming between the two components (Figure 4e). Additionally, NF positive nerve fibers extending from the DRG (GFP negative) or GMLT (GFP negative) were well ensheathed by MBP positive myelin‐forming oligodendrocytes (GFP positive, arrows) inside the WMLT region (Figure 4f,g). On the other hand, muscle cells differentiated from mouse myoblasts (C2Cl2) appeared to attract the growth of nerve fibers from neurons in the SCLT to grow and make contacts with them. Confocal microscopy suggested that NF positive nerve fibers extending from the SCLT (from GFP transgenic SD rats) established axonal bouton–like contacts (arrows) with α‐bungarotoxin (α‐BT) positive motor endplate–like structures in the desmin positive muscle cells (Figure 4h).

### 2.5 Transplantation of SCLT Improved Hind Limb Motor Function

**Figure 5:**
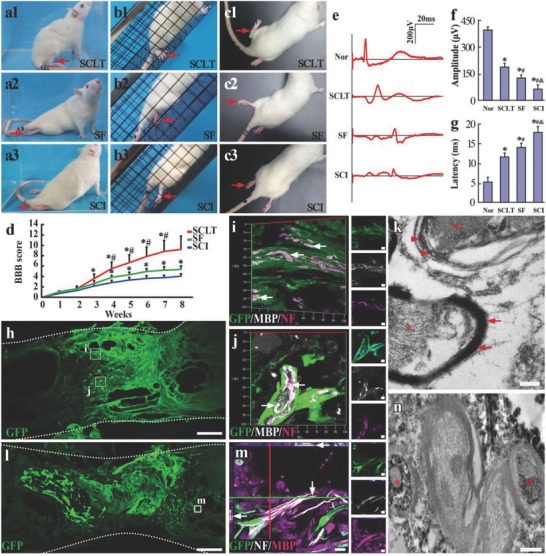
Behavior and electrophysiological improvement following the SCLT implantation and the integration of SCLT with the host spinal cord. a–d) Rats in the SCLT presented an overall improvement of hind limb motor function in the glass cube observation (a1), the inclined grid climbing test (b1), and the open field observation (c1), when compared with the Scaffold (SF) group (a2), (b2), (c2) or the transection spinal cord injury (SCI) group (a3), (b3), (c3). d) BBB scoring showed a significant increase in the hind limb motor function following the SCLT implantation, asterisks indicate statistical significance when compared with the SCI group (*p < 0.05 in (d)) and hash symbols indicate significant difference when compared with the SF group (^#^ *p* < 0.05 in (d)). e–g) Rats in the SCLT group have increased amplitudes and shortened latency of the CMEPs, resembling more similarities to those in the Normal (Nor) group (*p < 0.05, when compared with the Nor group; ^#^ *p* < 0.05 when compared with the SCLT group; ^&^ *p* < 0.05 when compared with the SF group). Asterisks indicate statistical significance when compared with the Nor group. h) GFP donor derived WMLT of a SCLT integrated with the host spinal cord 8 weeks after implantation. i,j) GFP positive donor cells expressed MBP and enwraped NF positive regenerating nerve fibers in the injury/graft site of spinal cord. k) Immunoelectron microscopy (IEM) showed that myelin sheath (stained by DAB, arrows) formed by GFP donor cell enwrapped an axon (asterisk). Host myelin sheath did not show DAB staining (arrowheads). l) GFP donor derived GMLT of a SCLT integrated with the host spinal cord 8 weeks after implantation. m) The GFP and NF double‐positive nerve fibers were surrounded by MBP positive cells. n) IEM showed that GFP positive axons (DAB labeled, asterisks) were enwrapped by myelin sheathes. Scale bars = 500 µm in panels (h) and (l), 10 µm in panels (i) and (j), 20 µm in panel (m), 200 nm in panels (k)–(n).

Immediately following the transected SCI with 2 mm spinal cord tissue removed in the adult SD rats, the SCLT or the gelatin sponge scaffold (SF) was implanted into the injury gap. Two months after the transplantation, the rats in the SCLT group showed significant hind limb motor function and electrophysiological presentation improvement compared with those in the control groups (the SF group and the SCI group). The body weight support capability of the hind limbs during immobile posture for rats in the SCLT group was noticeably better than those in the other two groups (**Figure** **5**a1–a3), suggesting a better preservation of muscular strength. Additionally, compared with those from the other two groups, rats from the SCLT group exhibited more frequent weight‐bearing stepping during mobile courses, such as grid climbing or locomotion in the open field (Figure 5b1–b3,c1–c3). Basso, Beattie, and Bresnahan (BBB) open‐field locomotor test was used to quantify the hind limb motor function for rats in the SCLT, SF, and SCI groups (*n* = 8 in each group). The results showed a distinctively improved motor function for rats in the SCLT group than those in the other two groups beginning at the 3rd week after SCLT transplantation. The BBB score for rats of the SCLT group was above 9 points at 8 weeks, indicating there was weight‐bearing locomotion (Figure 5d). As a widely used clinical evaluation, the peak and the latency of the cortical motor evoked potentials (CMEPs) are interpreted conventionally as major parameters reflecting the number of excited axons and the conduction velocity of the nerve, respectively. In the SCLT group, increased amplitude and shortened latency of cortical motor evoked potential (EP) were observed, suggesting a better repair for the motor pathway (Figure 5e–g).

Within 8 weeks of observation time, rats did not show any sign of distress, such as significant body weight loss, aggression, or vocalization when touched, or porphyrin staining following the implantation of SCLT.

### 2.6 SCLT Implant Conferred Targeted Structural Repair in Injured Spinal Cord

Eight weeks after transplantation, the collagen scaffold degraded, leaving with no identifiable debris in the injury/graft site as detected by H&E staining (Figure S3, Supporting Information). The SCLT implant was endowed good histocompatibility and tissue repair as evident by significantly reduced cavity formation in the injured spinal cord when compared with the SF and SCI groups (Figure S3, Supporting Information).

GFP donor cells were used to separately construct the WMLT module or the GMLT module of a SCLT, so as to evaluate the structural integration between the host tissue and the two components of the implant two months after transplantation. In the present study, we did not observe any significant donor cell migration outside of the implant. The fluorescence signals emitted from the GFP donor–derived WMLT were mainly detected at the peripheral region of the injury/graft of spinal cord (Figure 5h). The majority of the donor cells in this region maintained MBP expression (Figure 5I,j and Figure S4a, Supporting Information) and formed sheaths enwrapping the NF positive axons (Figure 5i,j). Immunoelectron microscopy (IEM) showed that the GFP immunopositive sheaths (labeled by 3,3′‐diaminobenzidine (DAB), arrows in Figure 5k) presented a myelin sheath feature enwrapping the axon (asterisk in Figure 5k). Taken together, the WMLT component of the SCLT showed robust capability of myelinating the nerve fibers at the injury/graft site of spinal cord, representing an expected white matter repair after SCI. The fluorescence signals for the GFP donor–derived GMLT concentrated at the central region of the injury/graft (Figure 5l). The majority of the donor cells in the GMLT region of a SCLT were Map2 positive neurons or GFAP positive astrocytes (Figure S4b, Supporting Information). Similar to the neurotransmitter profile in vitro, the donor cells in the GMLT region exhibited the expression of GAD67, Glu, and ChAT when transplanted into the injured spinal cord (Figure S4c–f, Supporting Information). The nerve fibers outgrowing from the donor neurons (GFP positive), together with the host regenerating ones (GFP negative), helped increase the innervation of the injury/graft site of spinal cord. Samples derived from the implanted SCLT showed that most of the nerve fibers of donor neurons made close contacts with MBP positive sheaths (Figure 5m). IEM confirmed that GFP immunopositive axons (DAB labeled, asterisks in Figure 5n) were enwrapped by myelin sheaths. The results suggested that the GMLT component of the SCLT helped partially restore the loss of neuronal population in the injured area after SCI. Thus, it can be concluded that transplantation of the SCLT has helped repair the white matter and the gray matter damage simultaneously two months after the SCI.

### 2.7 The Donor Neurons Structurally Integrated with the Host Neural Circuits

**Figure 6:**
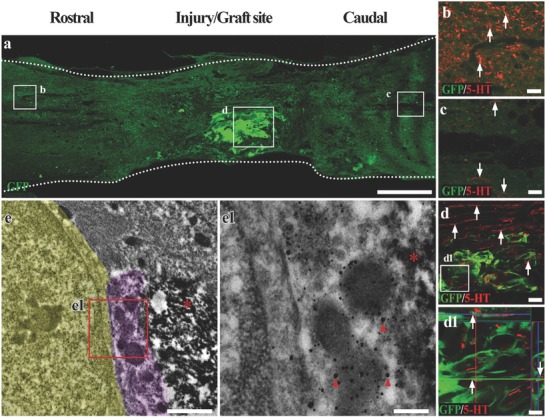
The donor neurons in the SCLT integrate with descending 5‐HT nerve fibers. a) A longitudinal section of spinal cord containing GFP donor derived GMLT of a SCLT. b,c) 5‐HT positive descending nerve fibers passed through the injury/graft site of spinal cord and extended several millimeters into the caudal area to the injury/graft site 8 weeks after SCLT implantation (c). d,d1) Most 5‐HT nerve fibers traveled longitudinally through the WMLT region, while some of them formed close contacts with the donor cells in the GMLT (arrows). e,e1) IEM showed that a 5‐HT nerve fiber (superimposed in light purple and labeled by nanogold particles, arrowheads in (e1)) formed close connection with GFP positive donor cells (DAB labeled, asterisks) and the host cell (superimposed in yellow). Scale bars = 500 µm in panel (a), 40 µm in panels (b)–(d), 20 µm in panel (d1), 1 µm in panel (e), 200 nm in panel (e1).

**Figure 7:**
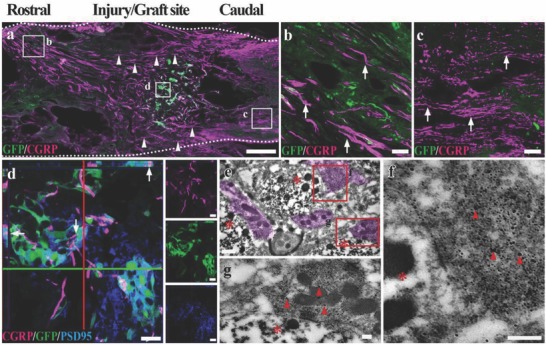
The donor neurons in the SCLT integrate with the ascending CGRP positive nerve fibers. a) A longitudinal section of spinal cord showed massive CGRP positive nerve fiber regeneration 8 weeks after the SCLT implantation. A large portion of CGRP nerve fibers traversed the WMLT region (arrowheads). b,c) CGRP signals can be observed in areas rostral (b) and caudal (c) to the injury/graft site of spinal cord. d) The CGRP positive nerve fibers (arrows) formed close contacts with PSD95 expressing GFP donor cells in the GMLT region of a SCLT. e–g) IEM showed that nanogold particle labeled CGRP nerve fibers (superimposed in light purple, arrowheads in (g) and (f)) closely contacted the transplanted cells (DAB labeled, asterisks). Scale bars = 500 µm in panel (a), 40 µm in panels (b) and (c), 20 µm in panel (d), 0.5 µm in panel (e), 200 nm in panels (g) and (f).

The 5‐HT positive nerve fibers descending from the brainstem severed after the transection injury were observed to regenerate and penetrate through the injury/graft site of spinal cord to areas several millimeters caudal to the injury/graft site in the SCLT group (**Figure** **6**a–c). On closer examination, the 5‐HT positive nerve fibers longitudinally penetrated the WMLT region (GFP negative zone, Figure 6d), suggesting the re‐establishment of passageway to the long descending supraspinal fibers. Some of the nerve fibers made contacts with the cells in the GMLT region, in a way similar to their segmental innervation in the physiological condition (GFP positive cells, Figure 6d). IEM confirmed the structural contacts between a 5‐HT positive nerve fiber (nanogold labeled, superimposed with light purple in Figure 6e,e1) and the adjacent donor cell (DAB positive, asterisk in Figure 6e,e1). In addition, the regeneration of sensory nerve fibers was assessed by calcitonin gene‐related peptide (CGRP) immunoreactivity (**Figure** **7**a). CGRP positive nerve fibers were widely detected in areas rostral (Figure 7b) and caudal (Figure 7c) to/in the injury/graft site in the SCLT group (Figure 7d). A large number of these nerve fibers made contacts with GFP/PSD95 double‐positive donor cells, indicating the potential of synapse forming. IEM showed many cell contacts established between the donor cells (DAB labeled, asterisks in Figure 7e) and the surrounding host CGRP positive cells (nanogold labeled, superimposed in light purple in Figure 7e). Some of the contacts displayed the features of a typical synapse such as the presence of round agranular vesicles (arrows in Figure 7f,g) in the presynpatic component (i.e., the CGRP positive nerve fiber terminal) and the focal membrane thickening in the postsynaptic component (GFP immunopositive donor cell labeled by DAB, Figure 7f,g).

Quantitative analysis showed that the number of NF, 5‐HT, and CGRP positive nerve fibers in the rostral and caudal areas to/in the injury site of spinal cord was significantly higher in the SCLT group, when compared with that in the SF or SCI groups (*p* < 0.01, *n* = 5 in each group; Figure S5, Supporting Information).

## 3 Discussion

Tissue engineering–based regenerative medicine has now entered a new era of applying implantable prebuilt tissue or organoid to repair tissue loss.^17^ Buoyed by the success in constructing functional neural networks from adult stem cells in vitro to repair SCI in our previous studies,^11^, ^18^ the present study has constructed a SCLT simulating the major cellular composition of the gray matter as well as the white matter of the spinal cord. When transplanted into the injured spinal cord, the SCLT integrated with the host tissue and exhibited targeted repair to the gray and white matter damage after injury. Thus, the modular assembly of SCLT may serve as a potential construct to help the structural and functional restoration after SCI.

Hydrogels are considered to be the most widely used bioscaffold to construct the organoids.[[qv: 5b,19]] In view of the weak mechanical property of the hydrogel, the volume of the organoid is often limited, and the maintenance of the scaffolding requires complex treatments.^20^ This property has made the hydrogel‐based organoid more suitable for an in vitro model, rather than an implant for in vivo treatment.[[qv: 6a,14]] On the other hand, the collagen sponge is mechanically stronger, and hence is easily tailored into column or ring shape. Additionally, the porous structure provides sufficient space for cell growth and differentiation; meanwhile it facilitates readily exchange of the nutrients and the wastes. As a result, cells residing in the SCLT presented a good viability and were able to survive in vitro up to one month. More importantly, the vast surface of the sponge offers the scaffold with ample space conducive for neuronal differentiation, nerve fiber extension, and establishment of intercellular contacts, including the synapses. The nonimmunogenic property of the collagen sponge makes it a safe biomaterial to use in the central nervous system as approved by the US Food and Drug Administration (FDA). The good mechanical property, along with its cytocompatibility and histocompatibility of the collagen sponge has allowed the construction of an optimal scaffold for SCLT.

CNTF plays an essential role in the differentiation and maturation of oligodendrocyte from its precursor OPCs.^21^ However, it is critical to predifferentiate NSCs into OPCs before applying CNTF so as to avoid astrocytic differentiation of NSCs as triggered by CNTF effect.^22^ Our previous studies focused on using NT‐3 and its receptor TrkC gene modification technique to promote neuronal differentiation of NSCs with synaptic transmission capability.^11^, ^23^ Here, we have adopted the same technique to construct the GMLT. By using this method, a mixture of neurons with the potentials to produce various neurotransmitters was generated, as evidenced by immunocytochemistry detection of GAD67, Glu, and ChAT. Electron microscopy showed synapse profiles forming between two neurons in the GMLT region. Along with the finding from FM1‐43 labeled vesicle releasing assay, our findings suggest that there may be synaptic transmission of impulses via neurotransmitters between two neurons. As further support, electrophysiology assessment of neurons in the GMLT region presented a mature neuron feature in bursting action potentials. Robust membrane potential fluctuations as recorded as EPSCs or IPSCs reflected the dynamic postsynaptic responses to the excitatory or the inhibitory neurotransmitter, respectively. These results indicated that the neurons in the GMLT region of a SCLT were synaptically connected to each other and formed a neuronal network capable of synaptic transmission.

The interactions between neurons and glial cells are vital for mutual maturation during development and maintenance of homeostasis.^24^ In this study, we also observed a dynamic interaction between the GMLT and the WMLT component when they were assembled into the SCLT. For example, although the singly cultured WMLT module exhibited a potential of forming myelin as evidenced by the detection of MBP immunoreactivity, there was a paucity of multilayered myelin sheath as observed under the electron microscopy, suggesting the structural immaturity of the oligodendrocytes. This might highlight the importance of axonal inputs in guiding the myelination process although the initiation of myelination could be independent from axon.^25^ Similarly, neurons in the singly cultured GMLT presented several immature features, as manifested by the synapse ultrastructure. When the SCLT was assembled, remarkably, multilayered myelin sheath was observed with axon being enwrapped in the WMLT region. Indeed, the delicate balance between the inhibitory and promoting molecules derived from axon is believed to be essential to guide the thickness of the myelin sheath.^26^ Meanwhile, the oligodendrocyte offers a variety of trophic factors that help the development and survival of neuron.^27^ Moreover, myelin aids axon metabolism^28^ and integrity.^29^ As we observed that focal membrane thickening, namely, the postsynaptic density, was evident at the synaptic contacts between two neurons in the GMLT region after being assembled into the SCLT. Moreover, aggregation of axons, dendrites, synapses containing at least two different types of vesicles, i.e., the small clear vesicles and the small dense‐core vesicles, and the surrounding glial cell processes, were common in the border area between the GMLT and the WMLT; such a configuration and ultrastructural features resemble the neuropil in the central nervous system (CNS). All this suggests that the assembly of the SCLT represents not only a structural combination of the WMLT module and the GMLT module, but also allows a dynamic integration of function between the two components that enhances the further maturation of each other. Therefore, the SCLT may serve as a platform for study of the interactions between the neurons and the glia cells in vitro.

After 14 days of culture, the SCLT shared many similarities to the normal adult spinal cord in terms of gene and protein expression as evaluated by Q‐PCR and Western blotting, respectively. The differences between these two entities lied in the expression level of some mature neuron markers, for example, the expression of SYP, encoding a compositional presynaptic protein, was observed highly expressed in the adult spinal cord at mRNA and protein levels. On the contrary, some immature/developmental markers, such as the tubb3, encoding class III β‐tubulin, whose expression levels were higher in the SCLT. This suggests that, despite the mature oligodendrocyte or neuron phenotypes and the functions observed, the SCTL might retain the potential for further development and maturation after 14 days of culture. This notion is supported by the findings from the coculturing of the SCLT with the organotypic DRG or muscular cells. The structural integration between the SCLT and DRG or muscle cells suggests that, like the developing spinal cord, the SCLT is able to establish new connections with the DRG, which represents the afferent inputs from the peripheral, and with the muscle cells, which may be regarded as the targets of the efferent nerve fibers. The intermediate stage of the SCLT between the immature and the mature stage would render it a promising application value when implanted into the injured spinal cord. This is because it is generally believed the immature cells adapt to the post SCI microenvironment better than the mature ones.[[qv: 8a,30]]

Two months after SCLT transplantation, donor cells, both in WMLT and GMLT, survived well and maintained oligodendroglial or neuronal phenotype similar to that observed in vitro. The strategy of transplantation of in vitro prebuilt SCLT consisting of differentiated cells is believed to increase the safety of donor cells^31^ and dodge the uncertainty of cell differentiation amid postinjury microenvironment when compared to direct transplantation of stem or progenitor cells.[[qv: 10a,32]] Following SCLT transplantation, the overall hind limb motor function was significantly improved, compared with the control groups; in fact, it was superior to that of what we reported before using NSC‐derived neural network transplantation (equivalent to the GMLT transplantation).^12^ Consistent with previous observation, the GMLT module of the SCLT was capable of making contacts with the host descending or ascending neural circuits that would potentially serve as “tissue engineering neuronal relay” to conduct signals passing through the injury/graft site of spinal cord. Furthermore, the WMLT module may provide an additional benefit in motor pathway repair. Indeed, the oligodendrocytes in the WMLT region were able to myelinate the nerve fibers in the injury/graft site of spinal cord. This could help repair the white matter loss and presumably contribute to the shortened latency as demonstrated in the present CMEP recording. Another feature worthy of note is that the WMLT region may offer inductive microenvironment for the regeneration of descending supraspinal nerve fibers, for example, exuberant 5‐HT nerve fibers were observed traversing the WMLT region and reinnervating the spinal cord segment caudal to the injury/graft site of spinal cord.

## 4 Conclusions

All in all, the assembly of the SCLT has realized a dynamic interaction and functional integration of the WMLT module and the GMLT module. When transplanted into the injured spinal cord, the WMLT and the GMLT regions of the SCLT exhibited targeted repairing to tissue loss of the white and gray matter after SCI. As a result, improved paralysis hind limb motor function was observed following the SCLT transplantation. Transplantation of the SCLT is expected to provide a novel therapeutic approach for the structural and functional repair in the spinal cord transected completely or missing a segment spinal tissue. In addition, it may be used as an in vitro platform for study of neural development and neuropharmacology.

## 5 Experimental Section

*NSCs and OPCs Culture and Identification*: NSCs were isolated from SD rats or GFP transgenic SD rats (Osaka University, Osaka, Japan), as described previously.^32^ Briefly, rats (1–3 days old) were anesthetized and the whole hippocampus was dissected and dissociated into single cell suspension. Cells were cultured in Dulbecco's modified Eagle's medium (DMEM)/F12 (1:1) supplemented with 1× B27 (Life Technologies, USA) and 20 ng mL^−1^ basic fibroblast growth factor (bFGF, Life Technologies, USA). Typically, cells were grown as neurospheres in suspension, which were passaged by mechanical dissociation approximately once each week. Nestin immunoreactivity was assessed and confirmed for all the neurospheres. Oligodendrocyte precursor cells (OPCs) were obtained according to the published protocols with slight modifications.^25^, ^33^ Briefly, NSCs at passage 2 were plated on polylysine‐coated culture dishes and cultured with DMEM/F12 containing 10 ng mL^−1^ platelet‐derived growth factor AA (PDGF‐AA, Life Technologies, USA), 10 ng mL^−1^ bFGF (Life Technologies, USA), 30 ng mL^−1^ triiodothyronine (T3, Sigma‐Aldrich), and 1% fetal bovine serum (FBS, Gibco) for 3 days. The cells were passaged when they reached 90% confluency, and were purified by differential digestion/adhesion technique. The cells were tested for the expression of NG2 (EMD Millipore). The purity of the OPCs used in all subsequent experiments was ≈80% NG2 positive.

*Modular Assembly of SCLT*: NSCs were transfected with lentivirus carrying a puromycin resistance gene and a neurotrophin‐3 (NT‐3) coding sequence (pLent‐EF1a‐NT‐3‐Flag‐CMV‐GFP‐P2A‐Puro) or its receptor TrkC (pLent‐EF1a‐TrkC‐Flag‐CMV‐GFP‐P2A‐Puro) gene (Vigene Biosciences). For OPC transfection, CNTF gene was delivered via lentivirus (pLent‐EF1a‐CNTF‐Flag‐CMV‐GFP‐P2A‐Puro, Vigene Biosciences). After 48 h of incubation, fresh medium containing 2 µg mL^−1^ puromycin was added for another 48 h to select the transfected cells.

For GMLT construction, a collagen sponge (BIOT Biology) scaffold was tailored into a short column (2 mm in diameter and 2 mm in length). A mixed cell suspension in 20 µL containing equal amount of NT‐3‐NSCs and TrkC‐NSCs (2 × 10^5^ for each scaffold) was dripped into the prewet scaffold. The GMLT module was maintained in DMEM/F12 (1:1) with 1× B27 supplement (Life Technologies, USA) and 1% FBS. For WMLT construction, a collagen sponge ring (3 mm in outer diameter, 2.0 mm in inner diameter, and 2 mm in length) was made by a biopsy punch. A total of 2 × 10^5^ CNTF–OPCs in 20 µL culture medium were seeded to the scaffold. The WMLT module was maintained in DMEM/F12 (1:1) with 1× B27 supplement and 1% FBS. The WMLT and GMLT modules were cultured separately for 7 days before they were assembled into the SCLT. The SCLT was maintained in DMEM/F12 (1:1) with 1× B27 supplement and 5% FBS for another 7 days.

*Organotypic Coculturing of SCLT with DRG or Muscular Cells*: DRGs for organotypic culture were established from 1 day old newborn SD rats. Dissection of DRGs from the spine column was done in ice‐cold DMEM/F12. The surrounding connective tissue and the adherent dura mater were removed. Three DRG blocks were placed on the top of each SCLT and were cultured for 7 days in DMEM/F12 medium containing 1× B27 supplement and 5% FBS with medium change every 2 days.

Mouse myoblasts (C2Cl2, a gift from Prof. H. Liao, Department of Human Anatomy, Southern Medical University, Guangzhou, China) were cultured in DMEM/F12 with 15% FBS at a density of 20 cells mm^−2^. When cells reached 80% confluency, they were induced to differentiate into muscle cells with DMEM/F12 plus 2% horse serum (Gibico). On day 7, SCLTs were placed above the muscular cells. The co‐culture system was maintained in SCLT culture medium. Vibration of the culture dish was minimized to allow extension of SCLT neurites to the muscle cells.

*Western Blotting and Quantitative Real‐Time PCR Analysis*: After 14 days of culturing, scaffolds (*n* = 3) from SCLT, GMLT, WMLT, or normal adult rat SC (positive control) were used for intracellular and extracellular protein extraction. Equal amount of proteins were loaded onto a 10% polyacrylamide gel. Proteins were separated by electrophoresis, followed by transferring them onto a polyvinylidene fluoride (PVDF) membrane. The membrane was incubated with primary antibodies at 4 °C overnight, followed by incubation with horseradish peroxidase (HRP)‐conjugated secondary antibodies. The bands were detected with an enhanced chemiluminescence (ECL) Western blotting substrate kit. The amount of glyceraldehyde 3‐phosphate dehydrogenase (GAPDH) protein was used as loading control.

Q‐PCR was used to compare the gene expression between SCLT (the SCLT group) and normal spinal cord (the SC group). Total cellular RNA in the SCLT or SC groups was extracted with TRIZOL reagent (Takara Bio Inc., Otsu, Japan). After reverse transcriptional reaction into complementary DNA (cDNA) (DRR037S, Takara Bio Inc., Otsu, Japan), Q‐PCR was carried out using Takara SYBR Premix Ex TaqTM mixture (DRR041S, Takara Bio Inc., Otsu, Japan), in a Bio‐Rad iCycler iQ5 PCR machine (BioRad). GAPDH was used as internal reference. Reaction protocol was as follows; 95 °C for 30 s, and 40 repeated cycles of 95 °C for 5 s, followed by 60 °C for 31 s. Each experiment was repeated for 3 times with a duplicate in each time. Primers for Q‐PCR are listed in Table S1 (Supporting Information).

*Whole‐Cell Patch Clamp*: The whole‐cell configuration was used to record the electrical activities of neurons in SCLT with a HEKA EPC amplifier 10 (HEKA Inc.). The results were analyzed by Patchmaster software (HEKA Inc.). Signals were filtered at 1 kHz and sampled at 5 kHz. The external solution contains 140 × 10^−3^ m NaCl, 5 × 10^−3^ m KCl, 2 × 10^−3^ m CaCl_2_, 1 × 10^−3^ m MgCl_2_, 10 × 10^−3^ m 4‐(2‐hydroxyethyl)‐1‐piperazineethanesulfonic acid (HEPES), and 10 × 10^−3^ m glucose (320 mOsm, pH set to 7.3 with Tris‐base). The patch electrodes had a resistance of 3–5 MW, when filled with pipette solution, containing 140 × 10^−3^ m CsCl, 2 × 10^−3^ m MgCl_2_, 4 × 10^−3^ m ethylene glycol‐bis(β‐aminoethyl ether)‐*N*,*N*,*N*′,*N*′‐tetraacetic acid (EGTA), 0.4 × 10^−3^ m CaCl_2_, 10 × 10^−3^ m HEPES, 2 × 10^−3^ m magnesium adenosine triphosphate (Mg‐ATP), and 0.1 × 10^−3^ m guanosine triphosphate (GTP). The pH was adjusted to 7.2 with Tris‐base, and the osmolarity was adjusted to 280–300 mOsm with sucrose. Electrophysiological recordings were performed at room temperature (22–24 °C). Mini postsynaptic currents (mPSCs) were counted and analyzed using Fitmaster (HEKA Inc.).

*Live‐Cell FM1‐43 and Calcium Imaging*: FM‐143 [(*N*‐3‐triethylammonmpropyl)‐4‐(4‐(dibutylamino) styryl)] (Life Technologies, USA) was used to evaluate synaptic vesicle releasing SCLT after high [K^+^] (50 × 10^−3^ m) stimulation.^34^ The first dose of high [K^+^] solution stimulated the recycling of endocytic synaptic vesicles that contained the FM1‐43. After 3 times of rinsing (15–20 min for each) with culture medium in the absence of FM1‐43, the endocytic/exocytotic activities were brought down to the basal level and the nonspecific labeling of cytoplasmic membrane was also eliminated while the synaptic vesicles kept the labeling of FM1‐43. After this, the second dose of high [K^+^] solution unloaded FM‐143 from the cells by inducing the depolarization process. Release of FM1‐43 labeled synaptic vesicles was captured by LSM780 confocal laser scanning system (Zeiss). A control experiment to assess nonspecific bleaching of fluorescence was performed simultaneously.

Live‐cell calcium imaging was performed using a LSM780 confocal laser scanning system (Zeiss), equipped with temperature and CO_2_ control module. For calcium imaging, Fluo‐4 (Life Technologies, USA) was prepared according to the manufacturer's instruction and was applied in the SCLT for 60 min in darkness. The supernatant was then removed and the SCLT was washed with a bath solution for 3 times. Imaging was performed at 494 nm excitation. Photos were taken every 20 s for 100 frames.[[qv: 5b,15]] Data analysis of calcium imaging was performed using HCimage Live 4.2.0. Region of interest (ROI) was manually selected and the mean fluorescence for each ROI was calculated at each time frame. Changes in fluorescence was enumerated as follows: Δ*F*/*F* = (*F* − *F*_basal_))/*F*_background_, where *F*_basal_ was the lowest mean fluorescence value of one frame and *F*_background_ was the average mean fluorescence of all frames. Neuropharmacological drugs (100 × 10^−3^ m glutamate, 50 × 10^−3^ m KCl, and 1 × 10^−3^ m TTX) were delivered by perfusion and kept for a 10 min incubation time before washing out.

*Surgery and SCLT Transplantation*: Three days before surgery, all animals were given cyclosporine A, intraperitoneally. Adult female SD rats (220–250 g, supplied by the Experimental Animal Center of Sun Yat‐sen University) were used in this study. Following laminectomy, a 2 mm cord segment including the associated spinal roots was completely removed at the T10 spinal cord level. After hemostasis was achieved, SCLT or collagen sponge scaffolds (the SCLT group or the SF group) were used to fill up the gap. The transection group (the SCI group) had 2 mm cord segment removed without filling in any biomaterials. Cyclosporine A (Novartis) was administrated once every day for two months. All experimental protocols and animal handling procedures were approved by the Animal Care and Use Committee of Sun Yat‐sen University, and were in compliance with the National Institutes of Health Guide for the Care and Use of Laboratory Animals.

*Assessment of Locomotor Performance*: Hind limb function of the rats was assessed weekly after surgery, using the BBB open‐field locomotor test,^35^ the glass cube locomotor function observation (the rat was put in a glass cube with 30 cm in height), and the inclined‐grid climbing test.^36^ BBB test was used to quantitatively evaluate the voluntary movements and the body weight support capability (*n* = 8 in the SCLT, SF, and SCI groups, respectively). Glass cube locomotor observation was used to generally observe the hind limb standing. Inclined‐grid climbing test was used to assess the accuracy of foot placement and coordination during locomotion. Two independent investigators who were blind to treatments determined the BBB scores.

*Electrophysiology*: At the end of the experiment, EP were recorded as described previously to assess the functional status of motor signal conduction (*n* = 8 in each group). Basically, following general anesthesia and exposure of the sciatic nerves and sensorimotor cortex (SMC), the electrodes (BL‐420E Data Acquisition Analysis System for Life Science, Taimeng, Chengdou, China) were connected to the sciatic nerve and SMC, respectively. The CMEPs were calibrated first, and then recorded as per the standardized protocols.^37^

*Perfusion and Tissue Preparation*: All rats were deeply anesthetized with 1% pentobarbital sodium (50 mg kg^−1^, intraperitoneally (i.p.)) and intracardically perfused with physiological saline containing 0.002% NaNO_2_ and 0.002% heparin, followed by 4% paraformaldehyde. After perfusion, the spinal cord was dissected, postfixed overnight in the same fixative, and dehydrated in 30% sucrose/phosphate buffered saline (PBS). Longitudinal sections of the selected spinal cord segments were cut at 25 µm thickness using a cryostat. All sections were stored at −30 °C until further processing.

*Immunofluorescence Staining*: Specific proteins were detected using immunofluorescence staining as described in the previous publications.^32^, ^37^ Briefly, the sections were incubated with primary antibodies mixed in 0.3% Triton X‐100 at 4 °C overnight, followed by the incubation with secondary antibodies. The slides were then examined under a fluorescence microscope. A summary of antibodies used is provided in Table S2 (Supporting Information).

*Ultrastructure Observations*: For SEM, scaffolds with cells were first washed 3 times with PBS, fixed in 2.5% glutaraldehyde for 90 min, dehydrated with a series of graded ethanol, and then freeze‐dried for 2 days. The dried samples were coated with gold and examined under a scanning electron microscope (Philips XL30 FEG).

For transmission electron microscopy (TEM), scaffolds were fixed with 2.5% glutaraldehyde at 4 °C for 1 h and postfixed with 1% osmic acid for 1 h. They were dehydrated through graded ethanol and embedded in Epon812 overnight, followed by polymerization at 60 °C for 48 h. Semithin sections (2 µm thickness) were cut on a Leica RM2065 microtome, mounted on glass slides, stained with toluidine blue (5%, in a borax solution) and mounted using neutral balsam before observation. Ultrathin sections (100 nm thickness) were cut, double stained with lead citrate and uranyl acetate, and examined under an electron microscope (Philips CM 10).

For IEM, rats were transcardially perfused with 0.1 mol L^−1^ of sodium phosphate buffer containing 187.5 units per 100 mL of heparin, followed by perfusion with 4% paraformaldehyde, 0.1% glutaraldehyde, and 15% saturated picric acid. The dissected spinal cord was postfixed overnight at 4 °C in fresh fixative and subsequently cut into 50 µm sagittal sections on a vibratome. To improve the penetration of antibodies, vibratome sections were transferred into cryprotectant solution containing 25% sucrose and 10% glycerol in 0.1 m PBS overnight at 4 °C, followed by a quick freeze–thaw in liquid nitrogen 3 times. After washing with PBS, the sections were treated for 1 h with 20% goat serum (Tris buffer, pH 7.4) to block nonspecific binding of the antibody. Sections were first incubated with primary antibodies (anti‐GFP combined with anti‐5‐HT or anti‐CGRP, *n* = 3) in 2% normal goat serum solution at 4 °C for 24 h, then incubated with secondary antibodies overnight at 4 °C, and postfixed in 1% glutaraldehyde for 10 min. The sections were detected by SABC‐DAB Kit and gold enhanced with Gold EnhanceTM EM Plus Kit (NanoProbe 2114, USA), osmicated, dehydrated, and embedded in Epon812. The Epon blocks were sectioned and examined under the electron microscope (Philips CM 10).

*Morphological Quantification*: For in vitro quantification of immunopositive cells, one in every ten of the whole series of sections from each scaffold was selected (*n* = 5 in each group). After immunostained with the respective markers, five areas (0.7 mm × 0.5 mm including four corners and one center) for each of the sections were chosen. The percentage of immunopositive cells were calculated by counting the total number of immunopositive cells. The numerical value obtained was then divided by the total number of GFP positive cells.

For in vivo quantification, areas that were 300 µm rostral or caudal to the injury/graft site, or that in the injury/graft site of each of the horizontal sections were scrutinized. One in every ten sections from each animal was processed; a total of five sections per rat were analyzed (*n* = 5 in each group). Three 0.7 mm × 0.5 mm areas for each of the sections cut through the rostral or caudal to the injury/graft site along with those for each of the sections cut through the injury/graft site were chosen. The percentage of immunopositive cells was calculated by counting the total number of immunopositive cells. The value obtained was then divided by the total number of GFP positive cells. For quantification of NF, 5‐HT, and CGRP positive nerve fibers, the immunopositive fibers with a length greater than 20 µm in the selected fields were counted.

*Statistical Analysis*: All statistical analyses were performed using the statistical software SPSS13.0. Data were presented as means ± standard deviation (S.D.). When three sets of data were compared, one‐way analysis of variance (ANOVA) with a least significant difference (LSD)‐*t* (equal variance assumed) or Dunnett's T3 (equal variance not assumed) was performed. A statistically significant difference was accepted at *p* < 0.05.

## Conflict of Interest

The authors declare no conflict of interest.

## Supporting information

Supplementary
