# Supplementary material for: A Modular Assembly of Spinal Cord–Like Tissue Allows Targeted Tissue Repair in the Transected Spinal Cord
Source: Adv Sci (Weinh). 2018 Jul 20;5(9):1800261. doi: 10.1002/advs.201800261 (PMC6145267; doi:10.1002/advs.201800261)
Supplement: Supplementary file 1 — Supplementary [file ADVS-5-1800261-s001.pdf]

Copyright WILEY-VCH Verlag GmbH & Co. KGaA, 69469 Weinheim, Germany, 2018.

ADVANCED SCIENCE
Open Access

Supporting Information

for *Adv. Sci.*, DOI: 10.1002/advs.201800261

A Modular Assembly of Spinal Cord–Like Tissue Allows Targeted Tissue Repair in the Transected Spinal Cord

Bi-Qin Lai, Bo Feng, Ming-Tian Che, Lai-Jian Wang, Song Cai, Meng-Yao Huang, Huai-Yu Gu, Bing Jiang, Eng-Ang Ling, Meng Li, Xiang Zeng, and Yuan-Shan Zeng**

Supporting Information

A Modular Assembly of Spinal Cord-Like Tissue Endows Targeted Tissue Repair in the Transected Spinal Cord

Bi-Qin Lai, Bo Feng, Ming-Tian Che, Lai-Jian Wang, Song Cai, Meng-Yao Huang, Huai-Yu Gu, Bing Jiang, Eng-Ang Ling, Meng Li, Xiang Zeng, Yuan-Shan Zeng**

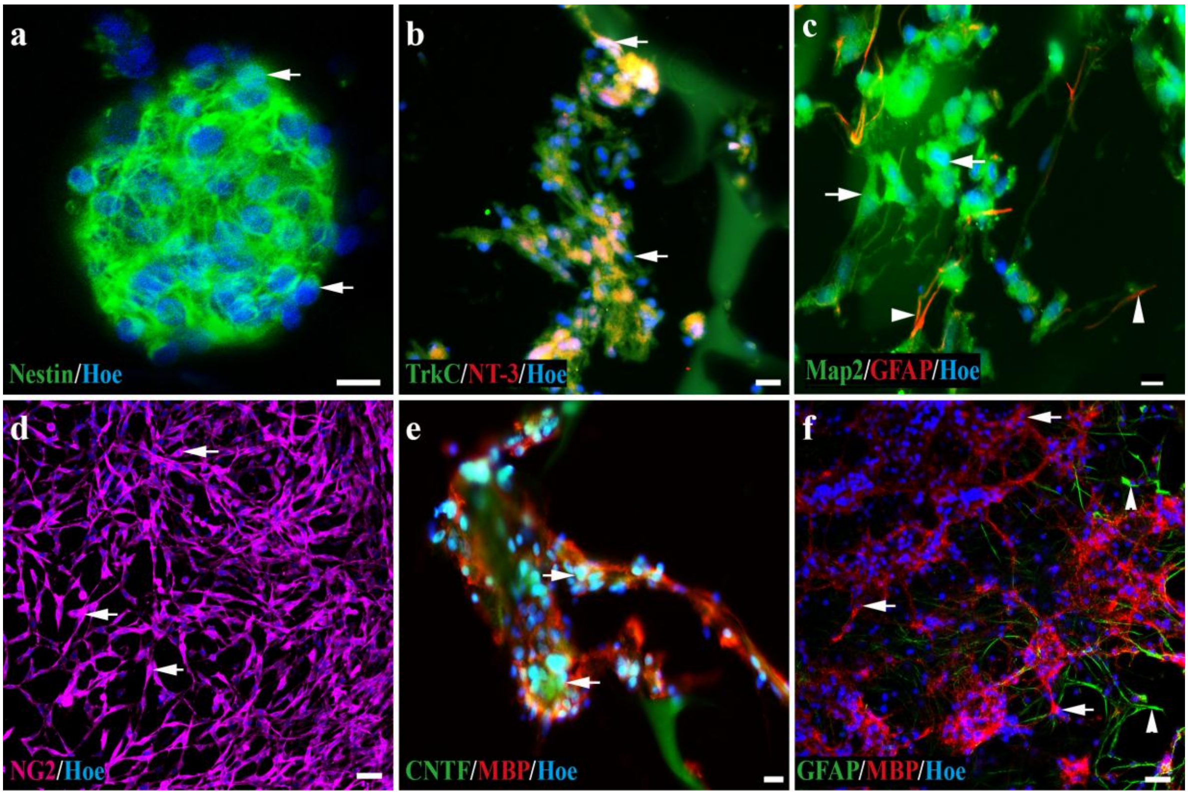

Figure S1. *In vitro* construction of SCLT. a) Neurospheres formed by Nestin positive NSCs (arrows). (b) Cells in a GMLT region expressing NT-3 and TrkC (arrows). c) Differentiation of cells in the GMLT region showed that most of the cells were Map2 positive (arrows) admixed with a few GFAP positive cells (arrowheads). d) After NSCs were induced and purified, 80% or more were NG2 positive cells (arrows). e) Cells in a WMLT region expressed CNTF and MBP (arrows). f) Most of cells differentiated into MBP positive cells (arrows) in the WMLT region, whereas a few of them differentiated into GFAP positive cells (arrowheads). Scale bars = 10 μm in panels (a) and (e), 20 μm in panels (b), (d) and (f).

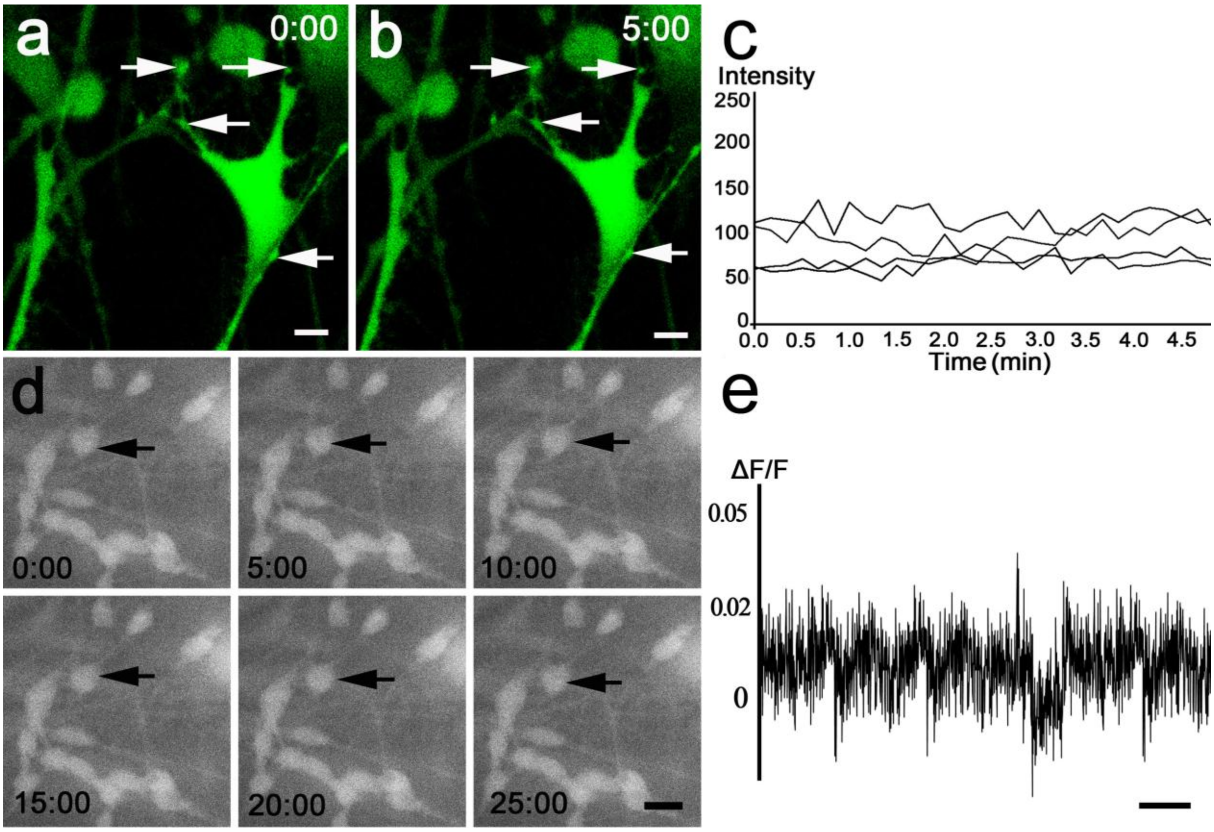

Figure S2. Functional assessment of the singly cultured GMLT module. a,b) Cells in the GMLT were observed to load pre-labeled FM1-43 dye (green) but not following membrane depolarization triggered by high [K^+] stimulation, as showed by the fluorescence intensity after the stimulation (c). d-e) Single-cell tracing of calcium surges revealed that cells (arrows in (d)) from the individually cultured GMLT displayed no spontaneous calcium surges (e) during Fluo-4 calcium live cell imaging. Scale bars = 10 μm in panels (a) and (b), 20 μm in panel (d), 100 seconds in panel (e).

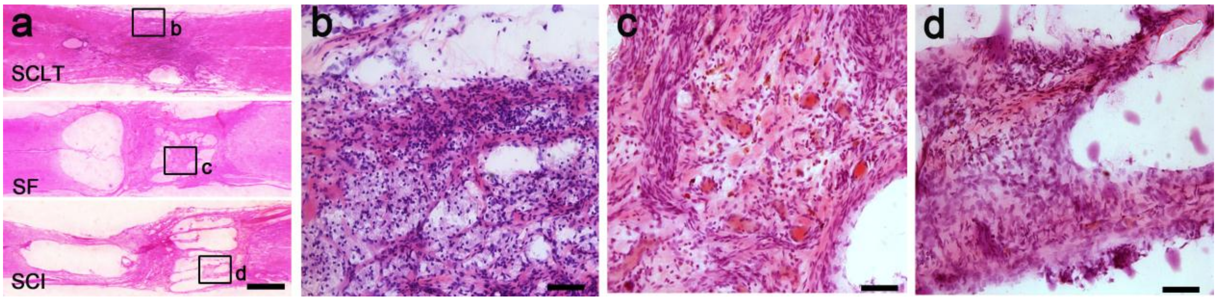

Figure S3. Hematoxylin and eosin (H&E) staining of spinal cord tissue. a) A longitudinal section of spinal cord showed that at 8 weeks post-injury, cystic cavities of varying sizes were more evident in areas rostral and caudal to the injury site as well as within the injury site in the SCI group compared with that in the SF and SCLT groups. b-d) Higher magnification images of the boxed areas in (a) at the epicenter areas of the injury/graft sites. Note the absence of identifiable residual materials in all these areas. Scale bars = 200 μm in panel (a), 20 μm in panels (b)-(d).

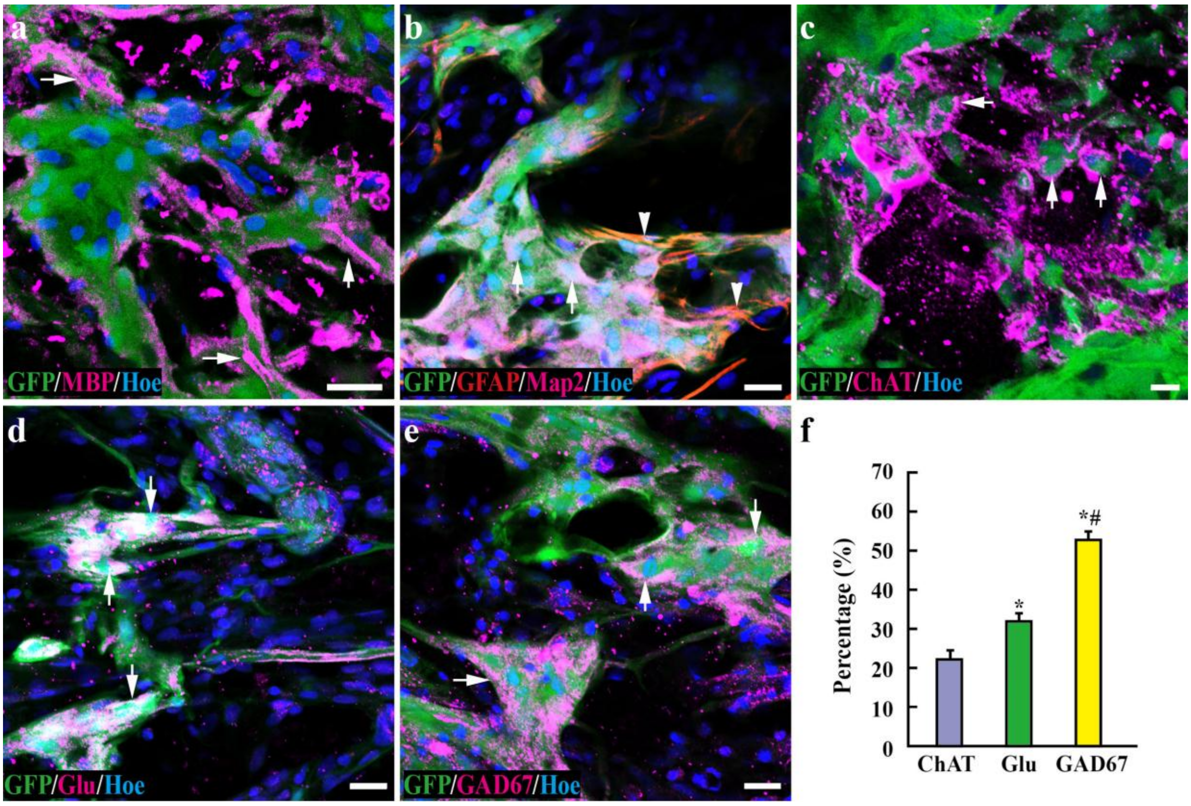

Figure S4. Cell differentiation of SCLT. a) 8 weeks after the transplantation of SCLT (WMLT cells were GFP positive and GMLT cells were GFP negative), GFP labeled WMLT cells expressed MBP (arrows). The ratio of MBP positive cells over GFP labeled cells was 57.70 ± 1.40%. b-e) 8 weeks after the transplantation of SCLT (WMLT cells were GFP negative and GMLT cells were GFP positive), a large number of GFP labeled GMLT cells expressed Map2 (arrows in (b)) and a small number of them expressed GFAP (arrowheads in (b)). The percentage of Map2 and GFAP positive cells among all GFP labeled cells was 60.20 ± 1.50% and 15.60 ± 1.80%, respectively. Some of the GFP labeled cells expressed ChAT (arrows in (c)), Glu (arrows in (d)) and GAD67 (arrows in (e)). f) Histogram showing the percentage of ChAT, Glu and GAD67 expressed by GFP labeled GMLT cells. Asterisks indicate statistical significance of Glu and GAD67 positive cells compared with ChAT positive cells ($^*P < 0.05$). Hash symbol indicates significant difference of GAD67 positive cells compared with Glu positive cells ($^\#P < 0.05$). Scale bars = 20 μm in panels (a)-(e).

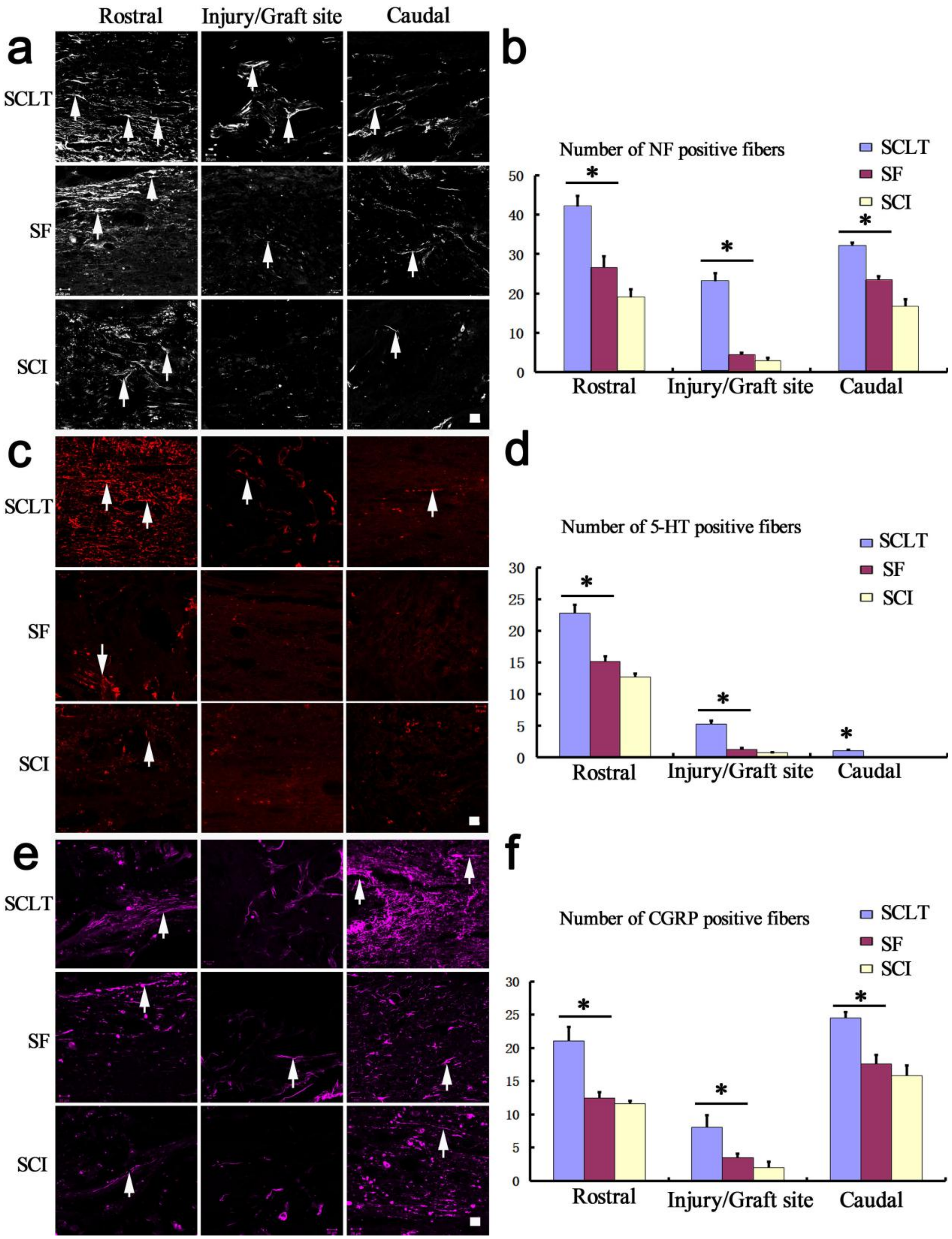

Figure S5. Distribution and number of NF, 5-HT and CGRP positive nerve fibers in the injured spinal cord at 8 weeks following surgery. a) NF positive fibers in the SCLT, SF, and SCI groups. b) Comparison of the number of NF positive fibers in the rostral and caudal areas

to/in the injury site of spinal cord among the three groups (*$P < 0.01$). c) 5-HT positive fibers in the SCLT, SF and SCI groups. d) Comparison of the number of 5-HT positive fibers in the rostral and caudal areas to/in the injury site of spinal cord among the three groups (*$P < 0.01$). e) CGRP positive fibers in the SCLT, SF and SCI groups. f) Comparison of the number of CGRP positive fibers in the rostral and caudal areas to/in the injury site of spinal cord among the three groups (*$P < 0.01$). Scale bars = 20 μm in panels (a), (c) and (e).

Table S1. Primers sequences for quantitative Real-Time PCR

Primers	Forward primer (5'…3')	Reverse primer (5'…3')
CALB2	GCATCTGCCGCTTGTTGAAG	CCCACCAGTGACCAGATCGA
SLC17A7	GCCATCATTGTCGCCAACTT	CCTTGCTGATCTCAAAGCCG
SYP	GCCCTGGCCACCTACATCTT	TGGCCCAGGCTGATGAACTA
MAPT	AGAAAGGCACATCCAATGCC	CTGTAGCCGCTTCGTTCTCC
Tubb3	GAGCCTGGAACCATGGACAG	ATAGTGCCCTTTGGCCCAGT
Pdgfra	ATGCCTTGAAAGCCACGTCA	GTTAAAGACGGCGCAGGTCA

Table S2. Primary and secondary antibodies

Antibodies	Species	Type	Dilution	Source (Catalog)
Neurofilament 200 (NF)	Mouse	Monoclonal IgG	1:1000	Sigma, St. Louis, USA (N0142)
Neurofilament 200 (NF)	Rabbit	Polyclonal IgG	1:400	Merck Millipore, Billerica, USA (N4142)
Synaptophysin (SYP)	Mouse	Monoclonal IgG	1:200	Sigma, St. Louis, USA (S5768)
Postsynaptic Density Protein 95 (PSD95)	Rabbit	Polyclonal IgG	1:800	Abcam, London, UK (ab18258)
Green Fluorescent Protein (GFP)	Rabbit	Polyclonal IgG	1:500	Merck Millipore, Billerica, USA (AB3080P)
Microtubule-associated protein 2 (MAP2)	Mouse	Monoclonal IgG	1:1000	Sigma, St. Louis, USA (M4403)
Choline Acetyltransferase (ChAT)	Rabbit	Polyclonal IgG	1:800	Merck Millipore, Billerica, USA (AB2219)
Glutamate (Glu)	Rabbit	Polyclonal IgG	1:500	Boster, Wuhan, China (BA0604-1)
glutamic acid decarboxylase 1 (GAD67)	Rabbit	Monoclonal IgG	1:500	Abcam, London, UK (ab213508)
TrkC	Goat	Polyclonal IgG	1:300	Sigma, St. Louis, USA (T2450)
Neurotrophin-3 (NT-3)	Rabbit	Polyclonal IgG	1:300	Sigma, St. Louis, USA (SAB1300907)
5-hydroxytryptamine (5-HT)	Rabbit	Polyclonal IgG	1:10000	Sigma, St. Louis, USA (SAB4501480)
Myelin Basic Protein (MBP)	Rabbit	Polyclonal IgG	1:400	Merck Millipore, Billerica, USA (AB980)
Glial fibrillary acidic protein (GFAP)	Rabbit	Polyclonal IgG	1:1000	Boster, Wuhan, China (PB0046)
Nestin	Rabbit	Monoclonal IgG	1:1000	Sigma, St. Louis, USA (SAB5500150)
CNTF	Rabbit	Polyclonal IgG	1:500	Abcam, London, UK (ab46172)
GAPDH	Mouse	Monoclonal IgG	1:10000	Abcam, London, UK (ab8245)

Calcitonin gene-related peptide (CGRP)	Rabbit	Polyclonal IgG	1:500	Boster, Wuhan, China (BA1572-1)
Desmin	Rabbit	Monoclonal IgG	1:800	Abcam, London, UK (ab32362)
Fibronectin	Rabbit	Polyclonal IgG	1:1000	Abcam, London, UK (ab2413)
Laminin	Rabbit	Polyclonal IgG	1:500	Abcam, London, UK (ab11575)
Chondroitin Sulfate (CS-56)	Mouse	Monoclonal IgG	1:1000	Abcam, London, UK (ab11570)
DyLigh 405 Goat Anti-Rabbit secondary antibody	Goat	Polyclonal IgG	1:500	Jackson ImmunoResearch, West Grove, USA (111-475-003)
Alexa 647 conjuncted anti rabbit secondary antibody	Goat	Polyclonal IgG	1:500	Jackson ImmunoResearch, West Grove, USA (100699)
Cy3 conjuncted anti rabbit secondary antibody	Goat	Polyclonal IgG	1:300	Jackson ImmunoResearch, West Grove, USA (711-165-162)
Cy3 conjuncted anti mouse secondary antibody	Goat	Polyclonal IgG	1:300	Jackson ImmunoResearch, West Grove, USA (115-165-146)
DyLigh 405 Goat Anti-Mouse secondary antibody	Goat	Polyclonal IgG	1:200	Jackson ImmunoResearch, West Grove, USA (115-475-146)
Goat Anti-Mouse (HRP)	Goat	Polyclonal IgG	1:5000	Abcam, London, UK (ab6789)
Goat Anti-Rabbit (HRP)	Goat	Polyclonal IgG	1:10000	Abcam, London, UK (ab6721)
α-Bungarotoxin (α-BT) Conjugates Alexa Fluor 555			1:500	Molecular Probe, (B35451)
